# Characterization of *oqxAB* in *Escherichia coli* Isolates from Animals, Retail Meat, and Human Patients in Guangzhou, China

**DOI:** 10.3389/fmicb.2017.01982

**Published:** 2017-10-13

**Authors:** Jing Wang, Chan-Ping Zhi, Xiao-Jie Chen, Ze-Wen Guo, Wu-Ling Liu, Juan Luo, Xin-Yi Huang, Li Zeng, Jia-Wei Huang, Ying-Bi Xia, Meng-Ying Yi, Teng Huang, Zhen-Ling Zeng, Jian-Hua Liu

**Affiliations:** Guangdong Provincial Key Laboratory of Veterinary Pharmaceutics Development and Safety Evaluation, College of Veterinary Medicine, South China Agricultural University, Guangzhou, China

**Keywords:** antimicrobial resistance, *Escherichia coli*, food safety, plasmids, PMQR

## Abstract

The purpose of this study was to investigate the prevalence and genetic elements of *oqxAB* among *Escherichia coli* isolates from animals, retail meat, and humans (patients with infection or colonization) in Guangzhou, China. A total of 1,354 *E. coli* isolates were screened for *oqxAB* by PCR. Fifty *oqxAB*-positive isolates were further characterized by pulsed-field gel electrophoresis (PFGE), multilocus sequence typing (MLST), S1-PFGE, genetic environment analysis, plasmid replicon typing, and plasmid sequencing. *oqxAB* was detected in 172 (33.79%), 60 (17.34%), and 90 (18.07%) *E. coli* isolates from animal, food, and human, respectively. High clonal diversity was observed among *oqxAB*-positive isolates. In 21 *oqxAB*-containing transformants, *oqxAB* was flanked by two IS*26* elements in the same orientation, formed a composite transposon Tn*6010* in 19 transformants, and was located on plasmids (33.3~500 kb) belonging to IncN1-F33:A-:B- (*n* = 3), IncHI2/ST3 (*n* = 3), F-:A18:B- (*n* = 2), F-:A-:B54 (*n* = 2), or others. Additionally, *oqxAB* was co-located with multiple resistance genes on the same plasmid, such as *aac(6*′*)-Ib-cr* and/or *qnrS*, which were identified in two F-:A18:B- plasmids from pigs, and *bla*_CTX−M−55_, *rmtB, fosA3*, and *floR*, which were detected in two N1-F33:A-:B- plasmids from patients. The two IncHI2/ST3 *oqxAB*-bearing plasmids, pHNLDF400 and pHNYJC8, which were isolated from human patient and chicken meat, respectively, contained a typical IncHI2-type backbone, and were similar to each other with 2-bp difference, and also showed 99% identity to the *Salmonella* Typhimurium *oqxAB*-carrying plasmids pHXY0908 (chicken) and pHK0653 (human patient). Horizontal transfer mediated by mobile elements may be the primary mechanism underlying *oqxAB* spread in *E. coli* isolates obtained from various sources in Guangzhou, China. The transmission of identical *oqxAB*-carrying IncHI2 plasmids between food products and humans might pose a serious threat to public health.

## Introduction

The efflux pump OqxAB, originally identified in the conjugative IncX1 plasmid pOLA52 from a porcine *Escherichia coli* isolate in 2003, belongs to the resistance-nodulation-division family, and is encoded by the *oqxA* and *oqxB* genes, which are located in the same operon (Sørensen et al., 2003; Hansen et al., 2004). OqxAB mediates resistance, or reduces susceptibility to multiple antimicrobials, including quinoxalines, chloramphenicol, trimethoprim, and quinolones, and is recognized as a plasmid-mediated quinolone resistance (PMQR) determinant (Hansen et al., 2007; Ruiz et al., 2012).

Recently, the *oqxAB* genes have been identified as the most prevalent PMQR genes in *E. coli* isolates from food-producing animals in China (Liu et al., 2012, 2013; Xu et al., 2015), as well as from animal-derived food products (Xu et al., 2014). This could be due to the widespread use of olaquindox as a growth promoter for pigs weighing below 35 kg and mequindox against enteropathogenic *E. coli* infections in swine and poultry (He T. et al., 2015). Our previous study demonstrated high *oqxAB* prevalence in *E. coli* isolates from animals, farm environment, and farm workers, and clonal transmission of *oqxAB*-carrying isolates between swine and farm workers was observed (Zhao et al., 2010). Additionally, *oqxAB* was observed to be relatively prevalent in *E. coli* isolates from pigs, ducks, chickens, meat (pork and chicken meat), and healthy humans (55.7, 40.6, 25.8, 16.2, and 7.2%, respectively; Yang T. et al., 2014). However, *oqxAB* has been rarely reported in human clinics and animals in Europe, where olaquindox has been banned as an animal feed additive since 1999 (Hansen et al., 2005), with detection rates of 0.46% in *E. coli* isolates from patients in the UK and Ireland (Ciesielczuk et al., 2013) and 1.62% from porcine *E. coli* strains from Denmark and Sweden (Hansen et al., 2005). Altogether, *oqxAB* might possibly be transmitted from animals to humans via the food chain or close contact, in China. Furthermore, IS*26* plays an important role in *oqxAB* dissemination (Hansen et al., 2004; He T. et al., 2015). IncHI2 plasmids have been recently demonstrated to mediate *oqxAB* spread in *S*. Typhimurium and *S*. Indiana among animals in China, as well as in clinical *S*. Typhimurium isolates in Hong Kong (Li et al., 2013; Wong et al., 2016). Thus, this study was aimed at determining the distribution and genetic elements (IS*26* and plasmids) of *oqxAB* among *E. coli* isolates from different sources (animals, animal-derived food products, and human clinics) in Guangzhou, China, determining the complete nucleotide sequence of two IncHI2 plasmids carrying *oqxAB* and comparing them with those previously reported from different sources in China, and outlining the possible routes of *oqxAB* transmission via the food chain.

## Materials and methods

### Bacterial strains

A total of 1,354 individual *E. coli* isolates were collected from Guangzhou, Guangdong province, China between July 2011 and May 2013, including 509 animal strains (372 pig and 137 chicken strains) isolated from fecal samples of healthy animals from one hog market and one live poultry market, 346 strains from retail meat (247 pork and 99 chicken meat samples) recovered from fresh or chilled pork and chicken samples purchased from supermarkets and farmers' markets, and 498 strains from patients (71 *E. coli* strains from urine samples from inpatients with urinary tract infection, 427 *E. coli* strains from fecal samples of inpatients and outpatients) from four hospitals. The samples were inoculated onto MacConkey agar, and suspected *E. coli* colonies (one isolate per sample) were selected and identified by standard biochemical testing. All the isolates were tested for the presence of *oqxAB* by PCR, and 100 amplified products were randomly selected for sequencing (Table S1).

### Antimicrobial susceptibility testing

All *oqxAB*-positive *E. coli* isolates were tested for their MICs of ampicillin, cefotaxime, amikacin, gentamycin, neomycin, apramycin, tetracycline, florfenicol, ciprofloxacin, olaquindox, colistin, and sulfamethoxazole/trimethoprim using the agar dilution or broth microdilution method (limited to colistin). Antimicrobial susceptibility tests were performed and evaluated according to the protocols recommended by M100-S25 of the Clinical and Laboratory Standards Institute (Wayne, PA, USA) (Clinical and Laboratory Standards Institute, 2015). Colistin (>2 mg/L), neomycin (>8 mg/L), and florfenicol (>16 mg/L) were interpreted according to the clinical breakpoints or epidemiological cutoff values of the European Committee on Antimicrobial Susceptibility Testing (EUCAST; https://mic.eucast.org/Eucast2/); apramycin (>16 mg/L) and olaquindox (>64 mg/L) were analyzed according to DANMAP 2012 and DANMAP98, respectively (http://www.danmap.org/Downloads/Reports.aspx). The *E. coli* reference strain ATCC 25922 was used as the quality control. Epi Info version 7.2 (CDC) was used to perform statistical analysis. Comparison of prevalence of *oqxAB* and antimicrobials resistance rates was conducted by the χ^2^-test. *P* < 0.05 were considered statistically significant.

### Pulsed-field gel electrophoresis and multilocus sequence typing (MLST)

The genetic diversity of 50 randomly selected *oqxAB*-positive *E. coli* isolates from animals (*n* = 21), food products (*n* = 9), and patients (*n* = 20) was characterized by multilocus sequence typing (MLST; http://mlst.warwick.ac.uk/mlst/dbs/Ecoli) and PFGE (Gautom, 1997). MLST data were aligned using ClustalW, and a phylogenetic tree was constructed by the neighbor joining algorithm using MEGA 7.0 (Kumar et al., 2008). PFGE patterns were compared and analyzed using BioNumerics (Applied Maths, Sint-Martens-Latem, Belgium) using a cut-off value of 90% to define PFGE clusters.

### Conjugation/transformation experiments and plasmid characterization

The 50 *oqxAB*-positive *E. coli* strains subjected to PFGE were further analyzed by performing conjugation experiments with streptomycin-resistant *E. coli* C600 as the recipient strain following a previously described protocol (Chen et al., 2007). Transconjugants were selected using 32 μg/mL olaquindox and 3,000 μg/mL streptomycin.

Transformation was conducted via heat-shock or electroporation using *E. coli* strain DH5α as the recipient (Hanahan, 1983; Dower et al., 1988). Plasmid DNA was extracted using the E.Z.N.A. Plasmid DNA Midi Kit (Omega, Norcross, GA, USA). Transformants were selected using MacConkey agar plates containing 16 μg/mL olaquindox.

The presence of *oqxAB* in the transconjugants/transformants was confirmed via PCR and sequencing. Other resistance genes, including *floR, bla*_CTX−M_, *rmtB*, and *fosA3* were also screened using the primers listed in Table S1. Antimicrobial susceptibility of all the transformants and *E. coli* DH5α recipient strain were determined using the agar dilution or broth microdilution method (limited to colistin). All the transformants were characterized by PCR-based replicon typing and were screened IncX plasmids as described previously (Carattoli et al., 2005; Johnson et al., 2012). Replicon sequence typing and plasmid double locus sequence typing were performed to further characterize IncFII and IncHI2 plasmids according to previously described protocols (García-Fernández and Carattoli, 2010; Villa et al., 2010). S1-PFGE (Barton et al., 1995), and Southern blot hybridization were performed to determine the number of plasmids and the sizes of *oqxAB*-carrying plasmids in all the transformants, using a non-radioactively labeled *oqxAB*-specific probe. The genetic context of *oqxAB* was determined by PCR mapping (Table S2). Ten transformants with a single *oqxAB*-carrying plasmid were further analyzed by restriction fragment length polymorphism (RFLP) using the endonuclease *ApaL*I, TZC215-1, and AHH13-1 were excluded since their sizes were significantly different from other *oqxAB*-carrying plasmids.

### Plasmid sequencing

Two IncHI2 plasmids, namely, pHNLDH400 from a patient with *E. coli* colonization and pHNYJC8 from chicken meat, were selected for sequencing. Plasmid DNA was purified from the transformants using QIAGEN® Plasmid Midi Kit according to the manufacturer's instructions (Qiagen, Hilden, Germany), and was completely sequenced by PacBio single-molecule real-time sequencing (RSII platform) (Pacific Biosciences, Menlo Park, CA, USA). Raw reads were introduced into the non-hybrid Hierarchical Genome Assembly Process. Analysis and annotation of the resulting sequences were performed using RAC (http://rac.aihi.mq.edu.au/rac/), ISfinder (https://www-is.biotoul.fr//), BLAST (http://blast.ncbi.nlm.nih.gov/Blast.cgi), RAST server (Aziz et al., 2008), and the Gene Construction Kit 4.0 (Textco BioSoftware, Inc., Raleigh, NC, USA). Plasmids pHNLDF400 and pHNYJC8 were compared with similar *oqxAB*-bearing IncHI2 plasmids pHXY0908 and pHK0653 obtained from *S*. Typhimurium, as well as our previously reported plasmids pHNSHP45-2 (Zhi et al., 2016), and pHNAH67 from *E. coli* isolate AHC67 (Yang X. et al., 2014) using BLASTn and BRIG (Alikhan et al., 2011). The IncHI2 plasmid R478 (GenBank accession number BX664015) served as the reference plasmid for annotation.

### Nucleotide sequences accession numbers

The nucleotide sequences of plasmids pHNLDF400 and pHNYJC8 have been deposited in the GenBank database under the accession numbers KY019258 and KY019259, respectively.

## Results and discussion

### Prevalence of *oqxAB* and antimicrobial susceptibility

Of 1,354 *E. coli* isolates examined in this study, *oqxAB* was detected in 322 (23.78%) *E. coli* isolates. Consistent with the results of our previous study (Yang T. et al., 2014), *E. coli* isolates from animal sources showed the highest *oqxAB* prevalence rate (172/509, 33.79%). However, *oqxAB* prevalence among the isolates of human origins (90/498, 18.07%) was slightly higher than those of food origins (60/346, 17.34%), contradictory to previous results, which was probably because we detected clinical *E. coli* isolates instead of strains from healthy volunteers. The *oqxAB*-positive isolates from patients were possibly selected under pressure exerted by antimicrobials, such as fluoroquinolones. However, *oqxAB* was previously reported to have low prevalence among clinical *E. coli* isolates in Europe (0.46%; Ciesielczuk et al., 2013), Korea (0.4%; Kim et al., 2009), Taiwan (6.05%; Kao et al., 2016), and mainland China (6.6%; Yuan et al., 2012; Zhao et al., 2015). Interestingly, the chicken isolates (51/137, 37.23%) showed a slightly higher *oqxAB* prevalence rate than the porcine isolates (121/372, 32.53%); similarly, the chicken meat isolates (22/99, 22.22%) showed higher *oqxAB* prevalence than the pork isolates (38/247, 15.38%).

**Figure 1:**
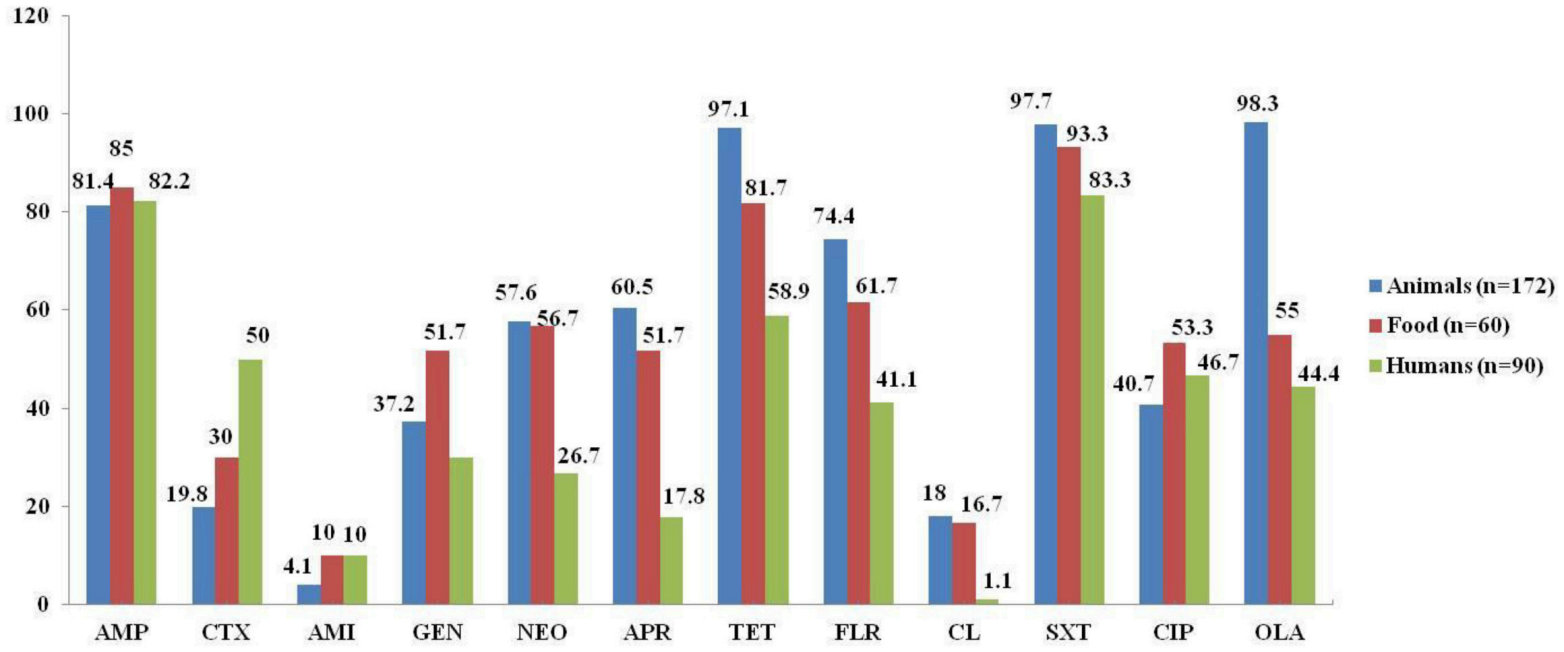
Comparison of antimicrobial resistance among *oqxAB*-positive *E. coli* isolates from food-producing animals, food products, and human patients. AMP, ampicillin; CTX, cefotaxime; AMI, amikacin; GEN, gentamycin; NEO, neomycin; APR, apramycin; TET, tetracycline; FLR, florfenicol; CL, colistin; SXT, sulfamethoxazole/trimethoprim; CIP, ciprofloxacin; OLA, olaquindox.

As shown in Figure 1, the isolates from food-producing animals showed the highest resistance rates against multiple antimicrobials, including neomycin (57.6%), apramycin (60.5%), florfenicol (74.4%), olaquindox (98.3%), and colistin (18.0%), which are used only in veterinary medicine in China (colistin was banned from animal feed in April 2017 in China), as well as tetracycline (97.1%) and sulfamethoxazole/trimethoprim (97.7%). It was followed by the isolates from food (56.7, 51.7, 61.7, 55.0, 16.7, 81.7, and 93.3%, respectively), and humans (26.7, 17.8, 41.1, 44.4, 1.1, 58.9, and 83.3%, respectively). However, the food isolates showed the highest resistance rate of 51.7% against gentamycin, which was considerably higher than the resistance rates of animal and human isolates (37.2 and 30%, respectively). In addition, the resistance rates against cefotaxime were significantly higher in the *oqxAB*-carrying isolates from humans (50%) than from animals (19.8%; *P* < 0.01) and food products (30%; *P* < 0.05), which could be explained by the relatively more frequent use of cephalosporins in clinical medicine.

### Molecular typing of *oqxAB*-positive *E. coli* isolates

The MLST analysis identified 21 different sequence types (STs) among 39 *oqxAB*-positive *E. coli* isolates (Table S3). The most commonly identified genotypes were ST10 (*n* = 10), followed by ST410 (*n* = 4) and ST744 (*n* = 3), whereas the isolates belonging to ST93 (*n* = 2), ST165 (*n* = 2), ST542 (*n* = 2), ST602 (*n* = 2), ST48, ST57, ST58, ST178, ST206, ST224, ST301, ST359, ST453, ST746, ST847, ST1421, ST3339, and ST6697 were also identified (Table S3). The remaining 11 *oqxAB*-positive *E. coli* were assigned to 11 novel STs (Table S3). In our study, the dominant ST10 clone, which has been frequently detected in animals, humans, food products, and the environment, and is responsible for the spread of multiple antibiotic resistance genes (Belmar Campos et al., 2014; Müller et al., 2016; Röderova et al., 2017), was detected in the isolates from animals, chicken meat, and patients. Additionally, other STs described in this study, such as ST410, ST744, ST93, ST48, and ST58, have been detected in various sources (Loncaric et al., 2013; Belmar Campos et al., 2014; Abraham et al., 2015; Falgenhauer et al., 2016; Röderova et al., 2017). The results of the phylogenetic analysis revealed the genetic relatedness of *oqxAB*-positive isolates (Figure S1).

**Figure 2:**
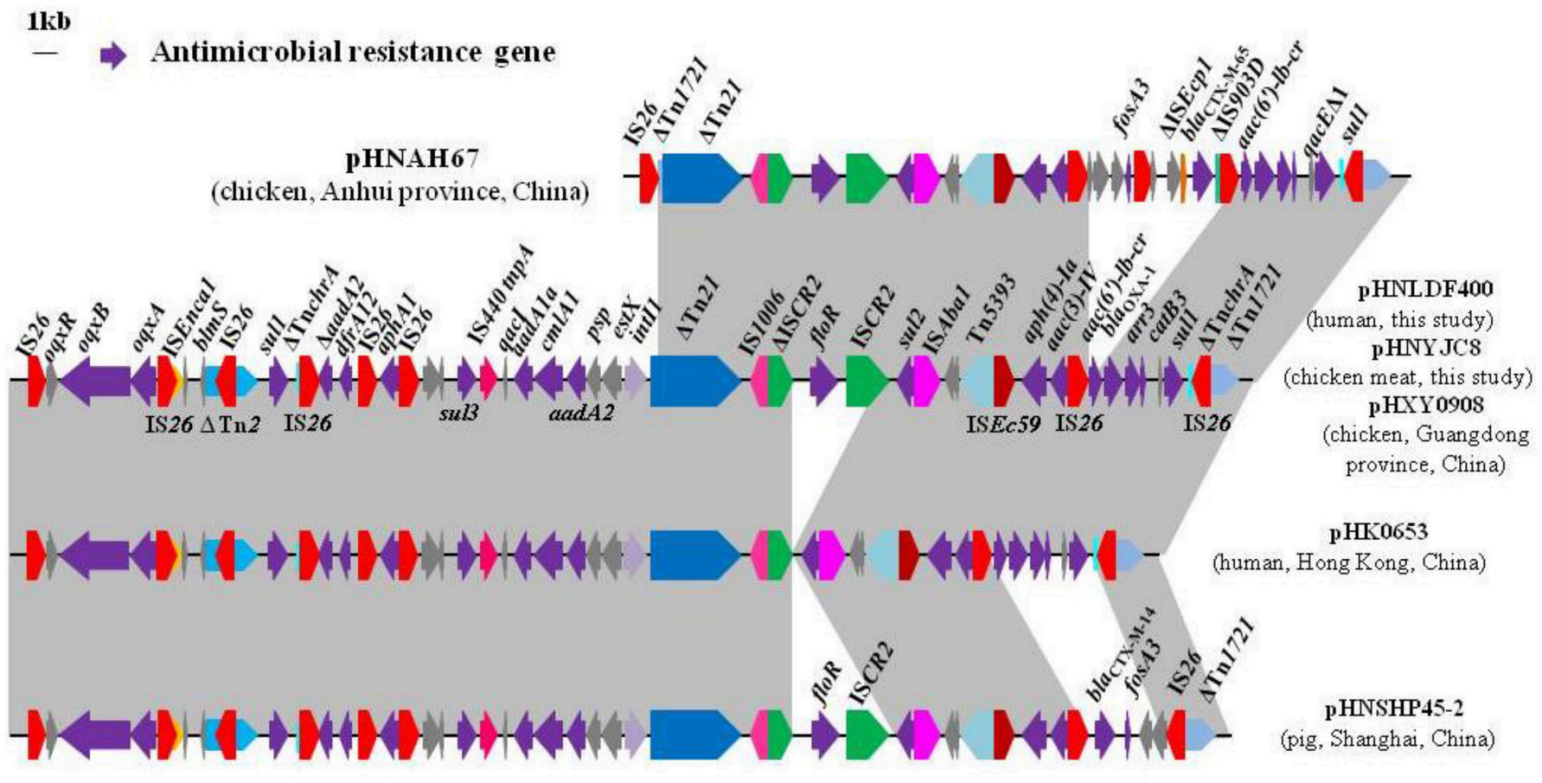
Comparison of the multidrug resistance regions of plasmids pHNLDF400 and pHNYJC8 with other similar IncHI2 plasmids. Arrows indicate the positions and directions of gene transcription. Regions with >99% homology are shaded in gray. ΔIndicates a truncated gene. The 1-kb distance scale is displayed in the upper left corner. Sequences were obtained from GenBank accession numbers pHNAH67, KX246266; pHXY0908, KM877269; pHK0653, KT335433; and pHNSHP45-2, KU341381.

Among the 50 *oqxAB*-positive *E. coli* isolates from different sources analyzed using *Xba*I-PFGE, 44 *oqxAB*-carrying *E. coli* isolates from animals (*n* = 19), animal-derived food products (*n* = 6), and patients (*n* = 19) exhibited 43 distinct PFGE patterns (Figure 2). Notably, the isolates belonging to the same STs identified in our study showed different PFGE patterns; however, the ST410 *E. coli* isolates BYMP20 and YZHF29, which were obtained from pork and human sources from different districts in Guangzhou within 12 km, showed similar PFGE patterns (E1 and E2; Figure 2).

MLST and PFGE demonstrated the molecular diversity of the *oqxAB*-positive *E. coli* isolates, and suggested that clonal transfer might not be the main mechanism underlying *oqxAB* dissemination among *E. coli* isolates in Guangzhou, China.

### Analysis of *oqxAB*-carrying plasmids

Surprisingly, none of the 50 randomly selected *oqxAB*-positive *E. coli* isolates transferred *oqxAB* to *E. coli* C600 via conjugation, which is consistent with the results reported for *S*. Typhimurium (Wong et al., 2014). Therefore, transformation assays were performed, and 21 transformants were obtained successfully. All the transformants were subjected to S1-PFGE and Southern blotting. The results demonstrated that the transformants carried one to four plasmids of various sizes. *oqxAB* was detected in plasmids with sizes ranging from ~33.3 to ~500 kb. Four transformants (SNJ23-1, SNJ41-1, ZYTM154-1, and LDHF159-1) showed two hybridization signals, whereas no hybridization signal was observed in SNX19-2 (Table 1). Thirteen transformants contained IncFII (*n* = 4), IncFIA (*n* = 2), IncFIB (*n* = 3), IncHI2 (*n* = 3), and IncN (*n* = 5) replicons. Notably, three transformants carried both IncN and IncFII replicons, which were classified as N1-F33: A-: B- (Table 1). Interestingly, the three transformants were obtained from three original *E. coli* isolates exhibiting different PFGE patterns from the same hospital, of which two carried only one *oqxAB*-bearing plasmid with similar sizes (Table 1 and Figure S2). Three IncHI2 plasmids from food product and patients were assigned to ST3 via plasmid double locus sequence typing (Table 1). PCR-based replicon typing could not determine the replicon types of the remaining eight transformants. Although rare, an untypable *oqxAB*-carrying plasmid was described previously (Liu et al., 2013). Ten transformants with single plasmid were selected for RFLP with *ApaL*I. Interestingly, two N1-F33:A-:B- plasmids from the patients of the same hospital (ZYTM118-1 and ZYTF32-1) showed identical patterns, as well as two F-:A18:B- plasmids from pigs (TZC152-6 and TZC212-1). Additionally, three ~76.8 *oqxAB*-bearing plasmids obtained from pig, chicken meat, and human, exhibited identical patterns (Figure S3).

**Table 1:** Characteristics of transformants carrying *oqxAB*.

**Strains^a^**	**Donor origin**	**Year**	**MLST**	**Other resistance genes**	**Other resistance patterns^b^**	**MIC (μg/mL)^c^**		**oqxAB-bearing Plasmid (Kb)^d^**	**Replicon type^e^**
						**CIP**	**OLA**		
DH5α						0.008	16		
WYMC1-1	Chicken meat	2011	ST10	*qnrS2, aac(6′)-Ib-cr, floR*,	AMP, GEN, TET, FFC, SXT	0.25	128	~76.8	ND
YJMC8-1	Chicken meat	2011	ST93	*qnrS2, aac(6′)-Ib-cr, floR*,	AMP, GEN, TET, FFC, SXT	0.25	128	~244.4 (4)	ST3-IncHI2
SNX19-2	Chicken	2012	ST542		AMP, GEN, TET, FFC, SXT	0.25	>256	ND (3)	N1-F-:A-:B54
SNJ11-1	Chicken	2012	ST10	*qnrS2, aac(6′)-Ib-cr, floR*	AMP, GEN, FFC	0.25	32	~104.5	F52:A-:B-
SNJ23-1	Chicken	2012	NEW	*qnrS2, aac(6′)-Ib-cr, floR*,	AMP, GEN, TET, FFC, SXT	0.25	>256	~350, ~33.3	ND
SNJ41-1	Chicken	2012	ST602		AMP, TET, SXT	0.03	256	~398.4, ~33.3	ND
SNJ43-1	Chicken	2012	ST6697			0.015	32	~76.8 (2)	ND
SNJ105-1	Chicken	2012	ST224	*floR*	AMP, GEN, TET, FFC, SXT	0.125	>256	~138.9	ND
SNJ113-6	Chicken	2012	ST178	*aac(6′)-Ib-cr, floR*	AMP, GEN, TET, FFC, SXT	0.125	>256	~80	ND
TZC48-1	Pig	2012	ST165	*qnrS2, aac(6′)-Ib-cr, floR*	AMP, TET, FFC, SXT	0.015	128	~104.5 (3)	F-:A-:B54
TZC152-6	Pig	2012	ST165	*aac(6′)-Ib-cr, qnrS2*	AMP, TET, SXT	0.25	256	~104.5	F-:A18:B-
TZC212-1	Pig	2012	NEW	*qnrS1*	AMP, TET, SXT	0.125	64	~104.5	F-:A18:B-
TZC215-1	Pig	2012	ST10	*qnrS1, aac(6′)-Ib-cr, floR*,	AMP, GEN, TET, FFC, SXT	0.125	64	~244.4	F-:A-:B54
TZC338-4	Pig	2012	ST10	*qnrS2, aac(6′)-Ib-cr, floR*,	AMP, GEN, TET, FFC, SXT	0.125	>256	~76.8	IncN1
AHH13-1	Hospital 1	2012	ST10		AMP, GEN, TET, SXT	0.06	64	~54.7	ND
ZYTF3-1	Hospital 1	2013	ST410	*qnrS2, aac(6′)-Ib-cr, floR*	AMP, GEN, TET, FFC, SXT	0.25	64	~76.8	ND
ZYTF32-1	Hospital 1	2013	ST58	*fosA3, rmtB, floR, bla*_CTX−M−55_,	AMP, CTX, AMI, GEN, FFC, SXT	0.015	64	~138.9	N1-F33:A-:B-
ZYTM118-1	Hospital 1	2013	NEW	*fosA3, rmtB, floR, bla*_CTX−M−55_,	AMP, CTX, AMI, GEN, FFC, SXT	0.015	64	~138.9	N1-F33:A-:B-
ZYTF154-1	Hospital 1	2013	NEW	*qepA, fosA3, rmtB, floR, bla*_CTX−M−55_,	AMP, CTX, AMI, GEN, TET, FFC, SXT	0.015	>256	~244.4, ~216.9	N1-F33:A-:B-
LDHF159-1	Hospital 2	2013	ST453	*aac(6′)-Ib-cr, floR*	AMP, GEN, TET, FFC, SXT	0.25	256	~500, ~244.4	ST3-IncHI2
LDHF400-1	Hospital 2	2013	ST57	*aac(6′)-Ib-cr, floR*,	AMP, GEN, TET, FFC, SXT	0.25	64	~244.4 (4)	ST3-IncHI2

a *Original isolates which cannot be typeable by PFGE are underlined. The transformants were designated as the name of their correspondent donor strains plus dash and number*.

b *AMP, ampicillin; CTX, cefotaxime; AMI, amikacin; GEN, gentamycin; TET, tetracycline; FFC, florfenicol; SXT, sulfamethoxazole/trimethoprim*.

c *CIP, ciprofloxacin; OLA, olaquindox*.

d *(n), number of plasmids in the transformant*.

e *ND, not determined*.

Sequences of the regions surrounding *oqxAB* were determined via PCR mapping and sequencing. *oqxAB* was flanked by two IS*26* elements in the same orientation, and formed a composite transposon Tn*6010* (IS*26*-*oqxA*-*oqxB*-*oqxR*-IS*26*) in 19 transformants. Additionally, one IS*26* element was present in upstream or downstream of *oqxAB* in the remaining transformants TZC338-4 and SNX19-2. Since its first identification in the plasmid pOLA52 from porcine *E. coli* in 2003 (Sørensen et al., 2003), Tn*6010* is believed to play a vital role in *oqxAB* transmission among Enterobacteriaceae isolates (Liu et al., 2013; He T. et al., 2015). Our results further support that horizontal transfer mediated by mobile elements seems to be the main mechanism for *oqxAB* transmission in *E. coli* from different sources.

The MICs of ciprofloxacin and olaquindox against the transformants were 2- to 32-fold (0.015–0.25 μg/mL) and 2- to > 32-fold (32 to >256 μg/mL) higher than the recipient *E. coli* DH5α strain (0.008 and 16 μg/mL), respectively. Furthermore, the co-transfer of resistance to ampicillin (*n* = 12), gentamycin (*n* = 10), tetracycline (*n* = 9), florfenicol (*n* = 9), sulfamethoxazole/trimethoprim (*n* = 11), cefotaxime (*n* = 3), and amikacin (*n* = 3) was observed in 12 transformants containing one plasmid. Multiple resistance genes were observed to coexist with *oqxAB* within the same plasmid accounting for the antimicrobial resistance; such as *bla*_CTX−M−55_, *rmtB, fosA3*, and *floR* were detected in two N1-F33:A-:B- plasmids from patients; *aac(6*′*)-Ib-cr* and/or *qnrS* were identified in both F-:A18:B- plasmids from pigs (Table 1). This allows for the co-selection of other genes conferring resistance to antibiotics that are routinely used in clinics, such as cephalosporins, aminoglycosides, and fosfomycin, or subject to selective pressure by quinolones, as well as quinoxalines used in food-producing animals. In turn, the co-selection of multiple drug resistance genes might facilitate further *oqxAB* transfer among Enterobacteriaceae isolates.

### Plasmid sequencing and comparative analysis

Two *oqxAB*-bearing IncHI2 plasmids in this study, namely pHNYJC8 from YJMC8 of food origin and pHNLDF400 from LDHF400 of human origin, were selected for sequencing. Plasmids pHNLDF400 and pHNYJC8 have sizes of 249,152 and 249,153 bp, respectively, and both have a GC content of 46.51%. They contain a typical IncHI2-type backbone, which encodes genes for plasmid replication, conjugative transfer, maintenance, and stability. Similar to other IncHI2 plasmids, such as pEC5207 (KT347600, porcine *E. coli*, China), pHNLDF400 and pHNYJC8 also carry a set of tellurite resistance determinants (*terZABCDEF*).

**Figure 3:**
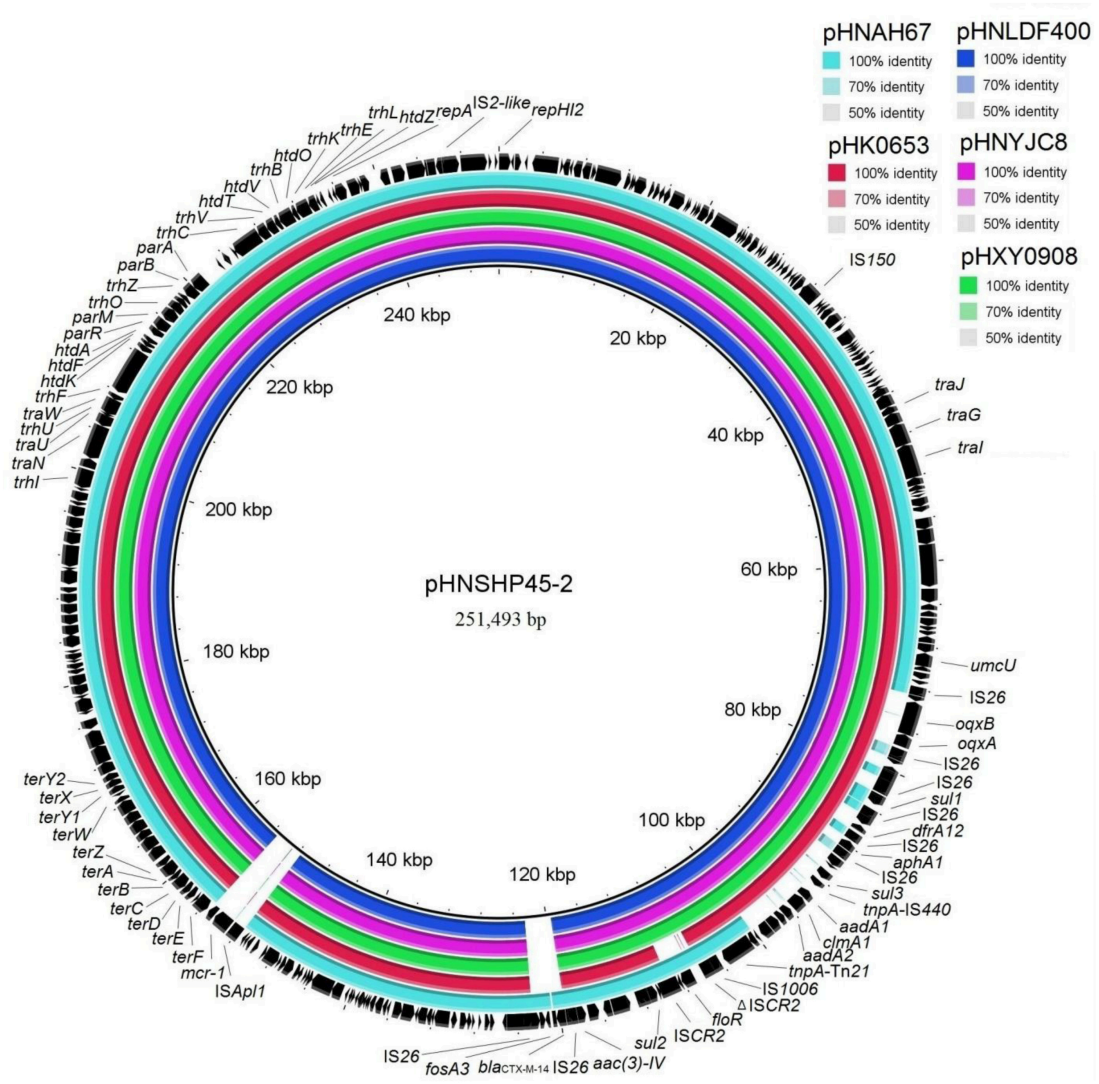
Sequence comparison of plasmids pHNLDF400 and pHNYJC8 with pHNSHP45-2 (GenBank accession number KU341381), pHXY0908 (KM877269), pHK0653 (KT335433), and pHNAH67 (KX246266) using BRIG. The reference sequence pHNSHP45-2 is shown in black.

Sequence comparisons demonstrated that pHNYJC8 and pHNLDF400, obtained from the *E. coli* clones ST93 and ST57, respectively, were identical with only 2-bp nucleotide difference, thereby confirming that the same *oqxAB*-carrying IncHI2 plasmid was transferred horizontally among *E. coli* isolates in human and food samples. Furthermore, both plasmids showed 99% identity to the previously sequenced *oqxAB*-bearing IncHI2 plasmids pHXY0908 from *S*. Typhimurium in chicken from China and pHK0653 from clinical *S*. Typhimurium in Hong Kong (Wong et al., 2016), except that a 4,284-bp fragment (*floR*-IS*CR2*) was absent in pHK0653 (Figures 2, 3), further suggesting that the same or similar *oqxAB*-carrying IncHI2 plasmids are transmitted among Enterobacteriaceae isolates from various sources in different geographic regions of China. Additionally, the backbones of pHNLDF400 and pHNYJC8 showed 99% identity to those of our previously sequenced colistin-resistant *mcr-1*-bearing plasmids pHNSHP45-2 (KU341381) obtained from porcine *E. coli* isolate SHP45 in Shanghai, China (Zhi et al., 2016) and pHNAH67 (KX246266) obtained from an *E. coli* isolate from chicken in Anhui province, China. However, the abovementioned plasmids contain distinct variable regions. pHNSHP45-2 contains a 3,376-bp fragment harboring *fosA3* and *bla*_CTX−M−14_. Contrastingly, in our study, both pHNLDF400 and pHNYJC8 contain a 4,713-bp segment harboring the |*aac(6*′*)-Ib-cr*|*bla*_OXA−1_|*catB3* |*arr3*|*qacE*Δ*1*|*sul|* cassette array, which is flanked by the 3′-conserved segment (3′-CS) and interrupted by a Tn*21*-like transposon Tn_chrA_, itself truncated by IS*26*. Most importantly, pHNSHP45-2 also harbors the colistin resistance gene, *mcr-1*, which is associated with IS*Apl1*, and is inserted in the plasmid backbone (Figure 3). Similarly, a 6,760-bp fragment containing *fosA3* and *bla*_CTX−M−65_ is identified in pHNAH67, whereas pHNLDF400 and pHNYJC8 contain a ~28.3-kb fragment with multiple resistance genes *oqxAB, sul1*, Δ*aadA2, dfrA12, aphA1, sul3, aadA1a, cmlA1*, and *aadA2*, which is absent in pHNAH67 (Figure 2). The absence/presence of these regions might be explained by IS*26*-mediated homologous recombination or replicative transposition (He S. et al., 2015). Furthermore, the *oqxAB*-associated composite transposon Tn*6010* and the upstream fragment (3,678-bp) containing ΔTn*2*, IS*26* (which interrupts the Tn*2* segment), and the bleomycin resistance genes *blms, orf63*, and ΔIS*Enca1* (which is truncated by IS*26* of Tn*6010*), were identical to the corresponding regions in the originally identified IncX1 *oqxAB*-carrying plasmid pOLA52, as well as pHXY0908, pHK0653, and pHNSHP45-2. The above mentioned results suggest that these plasmids may have originated from the same IncHI2-type plasmid, acquiring or losing various regions containing resistance genes, and spread among Enterobacteriaceae species in livestock, food products, and humans in different regions in China.

## Conclusion

In conclusion, our findings suggested that *oqxAB* dissemination among *E. coli* isolates from various sources could be due to horizontal transfer mediated by mobile elements, such as Tn*6010*, N1-F33:A-:B-, and IncHI2 plasmids. Identical *oqxAB*-carrying IncHI2 (ST3) plasmids were detected in the retail meat samples and human patients, similar to the previously described *oqxAB*-bearing IncHI2 plasmids pHXY0908 and pHK0653 from *S*. Typhimurium from chicken and patient. Thus, the transmission of similar *oqxAB* plasmids between animals, animal-derived food, and humans, and further human-to-human contact in communities and hospitals requires continued monitoring.

## Ethics statement

This study was carried out in accordance with the recommendation of ethical guidelines of South China Agricultural University. Individual written informed consent for the use of fecal or urine samples was obtained from all the patients and animal owners.

## Author contributions

JHL, ZZ, and JW conceived the study. CZ, XC, ZG, JL, JW, WL, XH, LZ, JH, YX, MY, and TH carried out the experiments. JW, CZ, and XC analyzed the data. JW wrote the manuscript. JHL and ZZ revised the manuscript. All authors read and approved the final manuscript.

### Conflict of interest statement

The authors declare that the research was conducted in the absence of any commercial or financial relationships that could be construed as a potential conflict of interest.
