# Supplementary material for: Characterization of oqxAB in Escherichia coli Isolates from Animals, Retail Meat, and Human Patients in Guangzhou, China
Source: Front Microbiol. 2017 Oct 13;8:1982. doi: 10.3389/fmicb.2017.01982 (PMC5645526; doi:10.3389/fmicb.2017.01982)
Supplement: Supplementary file 1 [file Table1.docx]

**Supplementary data**

**Table S1**. Primers used for PCR and DNA sequencing in this study

| Gene | Primer name | Sequence (5’ to 3’) | Reference |
| --- | --- | --- | --- |
| *oqxAB* | oqxAB-F | GTCCAGCGATAATCAGGC | 1 |
|  | oqxAB-R | GGTCTCGGCAATCACTTT | 1 |
| *floR* | floR-F | CTGAGGGTGTCGTCATCTAC | 2 |
|  | floR-R | GCTCCGACAATGCTGACTAT | 2 |
| *bla*_CTX-M-1_ | M1-F | CTTCCAGAATAAGGAATCCC | 3 |
|  | M1-R | CGTCTAAGGCGATAAACAAA | 3 |
| *bla*_CTX-M-9_ | M9-F | TGACCGTATTGGGAGTTTG | 3 |
|  | M9-R | ACCAGTTACAGCCCTTCG | 3 |
| *rmtB* | rmtB-F | ACATCAACGATGCCCTCAC | 4 |
|  | rmtB-R | AAGTTCTGTTCCGATGGTC | 4 |
| *fosA3* | fosA3-F | GCGTCAAGCCTGGCATTT | 5 |
|  | fosA3-R | GCCGTCAGGGTCGAGAAA | 5 |
| *qnrA* | qnrA-F | ATTTCTCACGCCAGGATTTG | 6 |
|  | qnrA-R | GATCGGCAAAGGTCAGGTCA | 6 |
| *qnrB* | qnrB-F | GATCGTGAAAGCCAGAAAGG | 6 |
|  | qnrB-R | ACGATGCCTGGTAGTTGTCC | 6 |
| *qnrC* | qnrC-F | GGGTTGTACATTTATTGAATCG | 7 |
|  | qnrC-R | CACCTACCCATTTATTTTCA | 7 |
| *qnrS* | qnrS-F | ACGACATTCGTCAACTGCAA | 6 |
|  | qnrS-R | TAAATTGGCACCCTGTAGGC | 6 |
| *qnrD* | qnrD-F | TTTTCGCTAACTAACTCGC | 8 |
|  | qnrD-R | GAAAGGATAAACAGGCAAAT | 8 |
| *aac(6’)-Ib* | aac(6’)-Ib-F | TTGCGATGCTCTATGAGTGGCTA | 9 |
|  | aac(6’)-Ib-R | CTCGAATGCCTGGCGTGTTT | 9 |
| *qepA* | qepA-F | CGGCGGCGTGTTGCTGGAGTTCTT | 10 |
|  | qepA-R | CCGACAGGCCCACGACGAGGATGC | 10 |

**References**

1. Wang, J., Guo, Z. W., Zhi, C. P., Yang, T., Zhao, J. J., Chen, X. J., et al. (2017). Impact of plasmid-borne *oqxAB* on the development of fluoroquinolone resistance and bacterial fitness in *Escherichia coli*. *J. Antimicrob. Chemother.* 72: 1293-1302. doi: 10.1093/jac/dkw576
2. Chen, S., Zhao, S., White, D. G., Schroeder, C. M., Lu, R., Yang, H., et al. (2004). Characterization of multiple-antimicrobial-resistant *salmonella* serovars isolated from retail meats. *Appl. Environ. Microbiol.* 70, 1-7.
3. Liu, J. H., Wei, S. Y., Ma, J.Y., Zeng, Z. L., Lü, D. H., Yang, G. X., et al. (2007). Detection and characterization of CTX-M and CMY-2 beta-lactamases among *Escherichia coli* isolates from farm animals in Guangzhou Province of China. *Int J. Antimicrob. Agents.* 29, 576-581.
4. Chen, L., Chen, Z. L., Liu, J. H., Zeng, Z. L., Ma, J. Y., Jiang, H. X. (2007). Emergence of RmtB methylase-producing *Escherichia coli* and *Enterobacter cloacae* isolates from pigs in China. *J. Antimicrob. Chemother.* 59, 880-885.
5. Hou, J., Huang, X., Deng, Y., He, L., Yang, T., Zeng, Z., et al. (2012). Dissemination of the fosfomycin resistance gene *fosA3* with CTX-M β-lactamase genes and *rmtB* carried on IncFII plasmids among *Escherichia coli* isolates from pets in China. *Antimicrob. Agents. Chemother.* 56, 2135-2138. doi: 10.1128/AAC.05104-11
6. Robicsek, A., Strahilevitz, J., Sahm, D. F., Jacoby, G. A., and Hooper, D. C. (2006). *qnr* prevalence in ceftazidime-resistant Enterobacteriaceae isolates from the United States. *Antimicrob. Agents. Chemother.* 2006; 50: 2872-2874.
7. Kim, H. B., Park, C. H., Kim, C. J., Kim, E. C., Jacoby, G. A., and Hooper, D. C. (2009). Prevalence of plasmid-mediated quinolone resistance determinants over a 9-year period. *Antimicrob. Agents. Chemother.* 53, 639-645. doi: 10.1128/AAC.01051-08
8. Zhao, J., Chen, Z., Chen, S., Deng, Y., Liu, Y., Tian, W., et al. (2010). Prevalence and dissemination of *oqxAB* in *Escherichia coli* isolates from animals, farmworkers, and the environment. *Antimicrob. Agents. Chemother.* 54, 4219-24. doi: 10.1128/AAC.00139-10
9. Park, C. H., Robicsek, A., Jacoby, G. A., Sahm, D., and Hooper, D. C. (2006). Prevalence in the United States of *aac(6’)-Ib-cr* encoding a ciprofloxacin-modifying enzyme. *Antimicrob. Agents. Chemother.* 50, 3953-3955.
10. Ma, J., Zeng, Z., Chen, Z., Chen, Z., Xu, X., Wang, X., Deng, Y., et al. (2009). High prevalence of plasmid-mediated quinolone resistance determinants *qnr*, *aac(6’)-Ib-cr*, and *qepA* among ceftiofur-resistant Enterobacteriaceae isolates from companion and food-producing animals. *Antimicrob. Agents. Chemother.* 53, 519-524. doi: 10.1128/AAC.00886-08

**Table S2.** Primers used for determining genetic environment of *oqxAB*

| **Region** | **Primer name** | **Sequence (5’ to 3’)** | **Reference** |
| --- | --- | --- | --- |
| IS*26*-*oqxA* | IS26oqxA*-*F | GCTGTTACGACGGGAGGAG | 1 |
|  | IS26oqxA*-*R | GGAGACGAGGTTGGTATGGA | 1 |
| *oqxA*-*oqxB* | oqxAB1-F | CAGGTGCTGTTCACGATA | 2 |
|  | oqxAB1-R | GTCGGAGACTGCTTCTGG | 2 |
|  | oqxAB2-F | GTCACCACCGTCACCTTC | 2 |
|  | oqxAB2-R | TTTTGCCTACCAGTCCCT | 2 |
|  | oqxAB3-F | GCGAAGAAAGACCTCCCTA | 2 |
|  | oqxAB3-R | CTGAGATCCGTCCACTCAA | 2 |
| *oqxB*-*oqxR* | oqxBR-F | CGCACCCGCAATAATCAGG | 2 |
|  | oqxBR-R | TGGTCGCAACGGCTCAATT | 2 |
| *oqxR*-IS*26* | oqxRIS26-F | GGTATGGCGAGCGAGGAC | 2 |
|  | oqxRIS26-F | GGAGATGCTGGCTGAACG | 2 |

**References**

1. Zhao, J., Chen, Z., Chen, S., Deng, Y., Liu, Y., Tian, W., et al. (2010). Prevalence and dissemination of *oqxAB* in *Escherichia coli* isolates from animals, farmworkers, and the environment. *Antimicrob. Agents. Chemother.* 54: 4219-4224.doi: 10.1128/AAC.00139-10.
2. Wang, J., Guo, Z. W., Zhi, C. P., Yang, T., Zhao, J. J., Chen, X. J., et al. (2017). Impact of plasmid-borne *oqxAB* on the development of fluoroquinolone resistance and bacterial fitness in *Escherichia coli*. *J. Antimicrob. Chemother.* 72: 1293-1302. doi: 10.1093/jac/dkw576

**Table S3**. Multilocus sequence typing of 50 selected *oqxAB*-positive isolates

| **Strain** | **Origin** | **Target gene** | | | | | | | **ST** |
| --- | --- | --- | --- | --- | --- | --- | --- | --- | --- |
|  |  | *adk* | *fumC* | *gyrB* | *icd* | *mdh* | *purA* | *recA* |  |
| SNJ11 | chicken | 10 | 11 | 4 | 8 | 8 | 8 | 2 | ST10 |
| TZC85 | pig | 10 | 11 | 4 | 8 | 8 | 8 | 2 | ST10 |
| TZC215 | pig | 10 | 11 | 4 | 8 | 8 | 8 | 2 | ST10 |
| TZC218 | pig | 10 | 11 | 4 | 8 | 8 | 8 | 2 | ST10 |
| TZC338 | pig | 10 | 11 | 4 | 8 | 8 | 8 | 2 | ST10 |
| TZC515 | pig | 10 | 11 | 4 | 8 | 8 | 8 | 2 | ST10 |
| WYMC1 | chicken meat | 10 | 11 | 4 | 8 | 8 | 8 | 2 | ST10 |
| AHH13 | patient | 10 | 11 | 4 | 8 | 8 | 8 | 2 | ST10 |
| AHH25 | patient | 10 | 11 | 4 | 8 | 8 | 8 | 2 | ST10 |
| CXHM186 | patient | 10 | 11 | 4 | 8 | 8 | 8 | 2 | ST10 |
| TZC178 | pig | 6 | 11 | 4 | 8 | 8 | 8 | 2 | ST48 |
| LDHF400 | patient | 6 | 31 | 5 | 28 | 1 | 1 | 2 | ST57 |
| ZYTF32 | patient | 6 | 4 | 4 | 16 | 24 | 8 | 14 | ST58 |
| YJMC8 | chicken meat | 6 | 11 | 4 | 10 | 7 | 8 | 6 | ST93 |
| ZYTF186 | patient | 6 | 11 | 4 | 10 | 7 | 8 | 6 | ST93 |
| TZC48 | pig | 10 | 27 | 5 | 10 | 12 | 8 | 2 | ST165 |
| TZC152 | pig | 10 | 27 | 5 | 10 | 12 | 8 | 2 | ST165 |
| SNJ113 | chicken | 10 | 4 | 4 | 8 | 8 | 8 | 2 | ST178 |
| SNJ55 | chicken | 6 | 7 | 5 | 1 | 8 | 18 | 2 | ST206 |
| SNJ105 | chicken | 6 | 4 | 33 | 16 | 11 | 8 | 6 | ST224 |
| TZC282 | pig | 78 | 27 | 5 | 10 | 12 | 8 | 2 | ST301 |
| LG09 | chicken meat | 43 | 41 | 15 | 90 | 11 | 8 | 6 | ST359 |
| BYMP20 | pork | 6 | 4 | 12 | 1 | 20 | 18 | 7 | ST410 |
| YZHF29 | patient | 6 | 4 | 12 | 1 | 20 | 18 | 7 | ST410 |
| ZYTF3 | patient | 6 | 4 | 12 | 1 | 20 | 18 | 7 | ST410 |
| LDHM95 | patient | 6 | 4 | 12 | 1 | 20 | 18 | 7 | ST410 |
| LDHF159 | patient | 99 | 6 | 33 | 33 | 24 | 8 | 7 | ST453 |
| SNX19 | chicken | 112 | 11 | 5 | 12 | 8 | 8 | 86 | ST542 |
| YZHF111 | patient | 112 | 11 | 5 | 12 | 8 | 8 | 86 | ST542 |
| SNJ41 | chicken | 6 | 19 | 33 | 26 | 11 | 8 | 6 | ST602 |
| YX42 | chicken meat | 6 | 19 | 33 | 26 | 11 | 8 | 6 | ST602 |
| YZHF179 | patient | 10 | 11 | 135 | 8 | 8 | 8 | 2 | ST744 |
| YZHF73 | patient | 10 | 11 | 135 | 8 | 8 | 8 | 2 | ST744 |
| HZMP34 | pork | 10 | 11 | 135 | 8 | 8 | 8 | 2 | ST744 |
| YJMC1 | chicken meat | 10 | 7 | 4 | 8 | 12 | 8 | 2 | ST746 |
| AHH32 | patient | 6 | 6 | 5 | 26 | 9 | 13 | 98 | ST847 |
| AHH66 | patient | 8 | 7 | 1 | 8 | 8 | 8 | 2 | ST1421 |
| PYMC1 | chicken meat | 10 | 11 | 4 | 358 | 274 | 8 | 42 | ST3339 |
| SNJ43 | chicken | 6 | 7 | 5 | 1 | 8 | 18 | 194 | ST6697 |
| SNX11 | chicken | 6 | 11 | 4 | 1 | 8 | 18 | 14 | NEW |
| SNJ127 | chicken | 6 | 11 | 4 | 8 | 8 | 111 | 2 | NEW |
| YJMC9 | chicken meat | 6 | 724 | 4 | 8 | 8 | 8 | 2 | NEW |
| ZYTM12 | patient | 8 | 7 | 1 | 8 | 8 | 109 | 6 | NEW |
| SNJ19 | chicken | 10 | 4 | 193 | 8 | 8 | 159 | 142 | NEW |
| SNJ23 | chicken | 10 | 7 | 4 | 8 | 8 | 1 | 2 | NEW |
| YZHF118 | patient | 10 | 11 | 4 | 8 | 452 | 13 | 2 | NEW |
| TZC212 | pig | 12 | 350 | 176 | 12 | 1 | 2 | 2 | NEW |
| ZYTM118 | patient | 64 | 23 | 358 | 91 | 307 | 7 | 2 | NEW |
| ZYTF154 | patient | 99 | 6 | 33 | 8 | 24 | 8 | 7 | NEW |
| LDHF158 | patient | 99 | 6 | 33 | 33 | 8 | 8 | 7 | NEW |

isolates designated with AHH, ZYT were obtained from hospital 1, LDH from hospital 2, CXH from hospital 3 and YZH from hospital 4.

**Figure S1**. Phylogenetic tree for 50 *oqxAB*-positive *E. coli* strains.


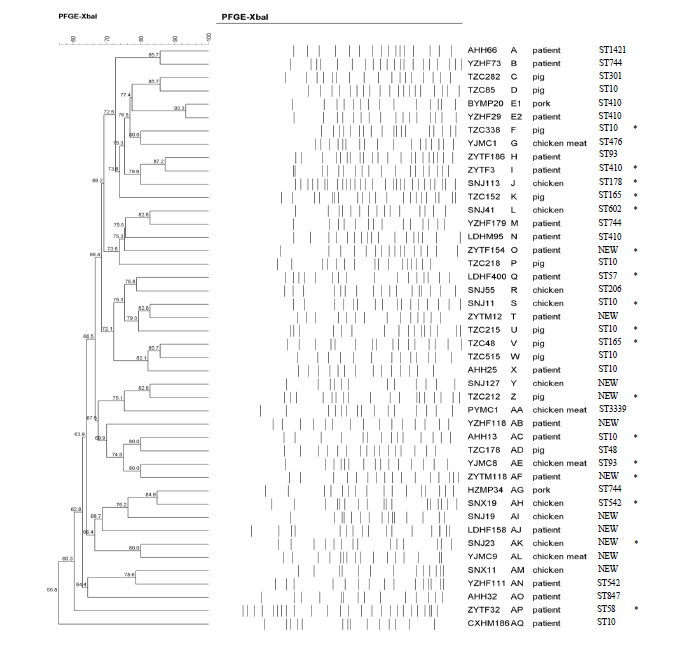


**Figure S2.** Dendrogram based on Unweighted Pair Group Method with Arithmetic Mean (UPGMA) clustering and PFGE profiles of selected *oqxAB*-positive isolates.

Isolates which the *oqxAB* genes can be transferred to the recipient by transformation are indicated by an asterisk.

1 2 3 M 4 5 6 7 M 8 9 10 M


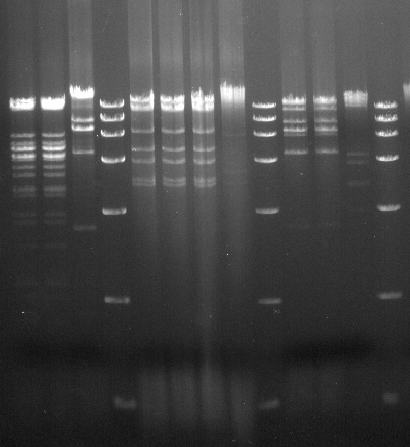


Figure S3 *ApaL*I restriction digestion proﬁles of plasmids harboring *oqxAB* genes from transconjugants with single plasmid.

Lanes 1–10: ZYTF32-1, ZYTM118-1, SNJ105-1, ZYTF3-1, WYMC1-1, TZC338-4, SNJ113-6, TZC212-1, TZC152-6, and SNJ11-1; Lane M: DL15000.
